# New challenges in diagnosis and treatment of chest pain in the patient with hyperuricemia

**DOI:** 10.1093/rap/rkaf134

**Published:** 2025-12-01

**Authors:** Yubing Zhang, Dongming Han, Yuhua He, Xiaoyao Chang, Ping Lu, Tao Zhang, Yanlong Jia, Hongqin Zhuang, Zichun Hua

**Affiliations:** State Key Laboratory of Pharmaceutical Biotechnology and Department of Neurology of Nanjing Drum Tower Hospital, School of Life Sciences and Affiliated Hospital of Nanjing University Medical School, Nanjing University, Nanjing, China; Department of Magnetic Resonance, First Affiliated Hospital of Xinxiang Medical University, Weihui, China; Department of Magnetic Resonance, First Affiliated Hospital of Xinxiang Medical University, Weihui, China; State Key Laboratory of Pharmaceutical Biotechnology and Department of Neurology of Nanjing Drum Tower Hospital, School of Life Sciences and Affiliated Hospital of Nanjing University Medical School, Nanjing University, Nanjing, China; Department of Oncology, First Affiliated Hospital of Xinxiang Medical University, Weihui, China; Faculty of Pharmaceutical Sciences, Xinxiang Medical University, Xinxiang, China; Faculty of Pharmaceutical Sciences, Xinxiang Medical University, Xinxiang, China; State Key Laboratory of Pharmaceutical Biotechnology and Department of Neurology of Nanjing Drum Tower Hospital, School of Life Sciences and Affiliated Hospital of Nanjing University Medical School, Nanjing University, Nanjing, China; State Key Laboratory of Pharmaceutical Biotechnology and Department of Neurology of Nanjing Drum Tower Hospital, School of Life Sciences and Affiliated Hospital of Nanjing University Medical School, Nanjing University, Nanjing, China; Faculty of Pharmaceutical Sciences, Xinxiang Medical University, Xinxiang, China; Changzhou High-Tech Research Institute of Nanjing University and Jiangsu Target Pharma Laboratories, Changzhou, China

A 27-year-old man presented to our outpatient department with a 3-day history of acute anterior chest pain. He had a history of gout and was first diagnosed with hyperuricemia (serum uric acid level 518 μmol/l) at the age of 24. In the days leading up to this visit, he experienced daily chest pain that progressively worsened from morning to night. The pain was sharp and localized over the costal cartilage, especially on palpation.

Physical examination revealed no significant abnormalities in the lungs or heart and no visible redness or swelling was observed over the chest wall (Fig. 1A). Laboratory tests showed a mildly elevated C-reactive protein level (1.76 mg/l). Musculoskeletal ultrasound revealed dot-like echogenic foci on the costal cartilage (Fig. 1B). Dual-energy computed tomography (DECT) of the chest wall demonstrated green colorization along the costochondral cartilage, indicating the presence of monosodium urate crystals (Fig. 1C). However, DECT of the hands and feet showed no crystal deposition (Fig. 1D).

**Figure 1:**
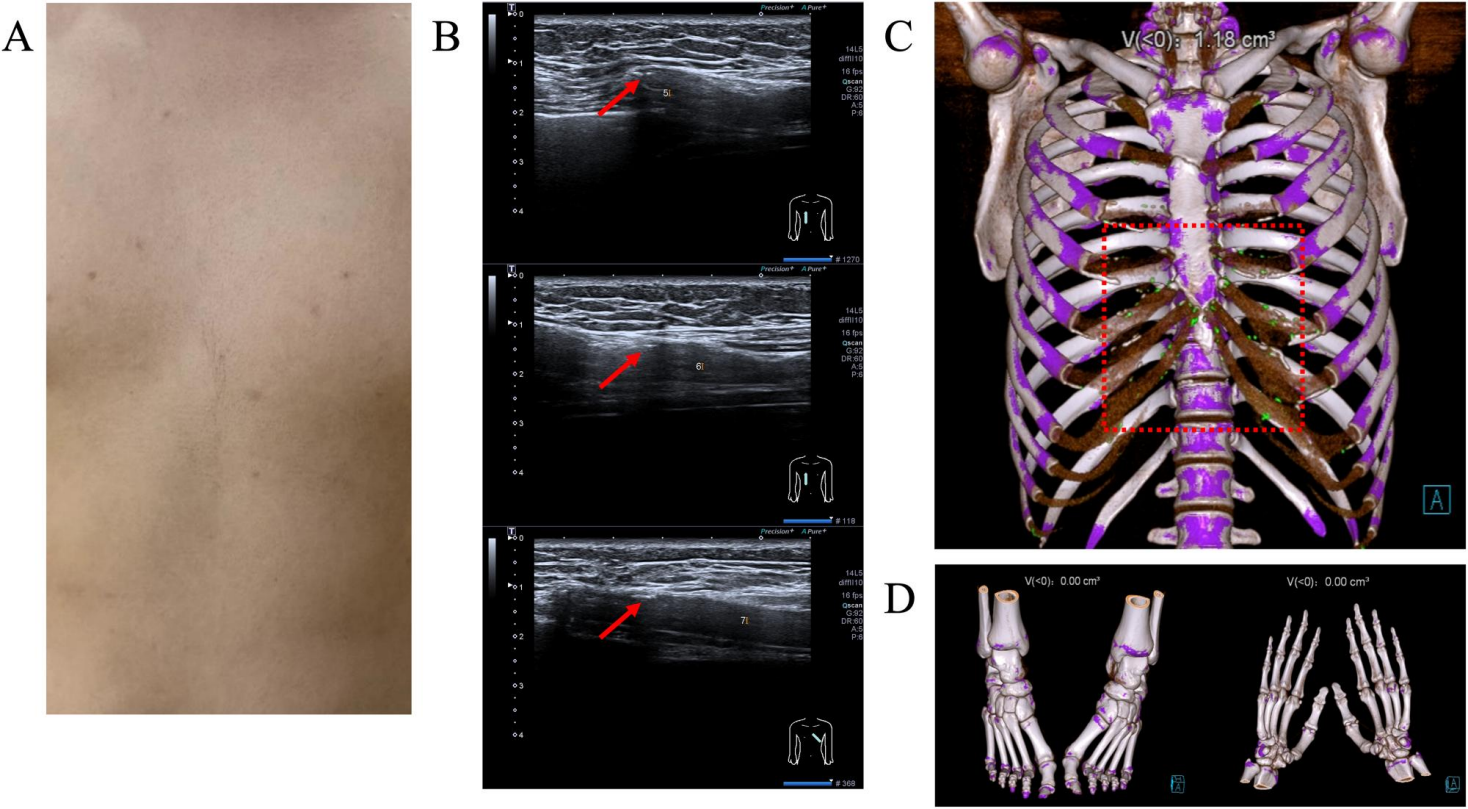
The imaging examination results of the patient: **(A)** chest photograph, **(B)** costal cartilaginous musculoskeletal ultrasonography, **(C)** chest DECT image and **(D)** DECT examination of both hands and feet

The patient was treated with etoricoxib (120 mg/day) and benzbromarone (100 mg/day), resulting in rapid pain relief within 2 days. Compared with allopurinol and febuxostat, benzbromarone demonstrates a faster absorption efficiency and a higher rate of uric acid reduction. Furthermore, there are fewer reports of adverse effects related to benzbromarone in Asian populations. Based on the evidence, we chose benzbromarone for treatment of this patient. Further documentation of similar clinical presentations and outcomes may help improve our understanding of the diagnosis and management of costochondral gout in patients. Detailed diagnosis and treatment records are presented in the supplementary materials, available at *Rheumatology Advances in Practice* online.

## Supplementary Material

rkaf134_Supplementary_Data

## Data Availability

The data underlying this article are available in the article and in its online supplementary material.

