# Supplementary material for: New challenges in diagnosis and treatment of chest pain in the patient with hyperuricemia
Source: Rheumatol Adv Pract. 2025 Dec 1;10(1):rkaf134. doi: 10.1093/rap/rkaf134 (PMC12758114; doi:10.1093/rap/rkaf134)
Supplement: rkaf134_Supplementary_Data [file rkaf134_supplementary_data.zip › SM Diagnosis and treatment records.docx]

# Supplementary material

# New challenges in diagnosis and treatment of chest pain in the patient with hyperuricemia

Yubing Zhang ^1^, Yuhua He ^2^, Xiaoyao Chang ^1^, Ping Lu ^3^, Tao Zhang ^4^, Yanlong Jia ^4^, Dongming Han ^2*^, Hongqin Zhuang ^1*^ and Zichun Hua ^1,4,5*^

Corresponding author: Zichun Hua, Email: [hzc1117@nju.edu.cn](mailto:huazc@nju.edu.cn)

Hongqin Zhuang, Email: [hqzhuang@nju.edu.cn](mailto:hqzhuang@nju.edu.cn)

Dongming Han, Email: [625492590@qq.com](mailto:625492590@qq.com)

**Affiliation**

1. The State Key Laboratory of Pharmaceutical Biotechnology and Department of Neurology of Nanjing Drum Tower Hospital, School of Life Sciences and The Affiliated Hospital of Nanjing University Medical School, Nanjing University, Nanjing, 210023, China

2. Department of Magnetic Resonance, The First Affiliated Hospital of Xinxiang Medical University; Weihui, 453100, China

3. Department of Oncology, The First Affiliated Hospital of Xinxiang Medical University; Weihui, 453100, China

4. Faculty of Pharmaceutical Sciences, Xinxiang Medical University; Xinxiang, 453003, China

5. Changzhou High-Tech Research Institute of Nanjing University and Jiangsu Target Pharma Laboratories Inc., Changzhou 213164, China

# Diagnosis and treatment details

The patient reported a medical examination at the hospital three weeks before the onset of the disease, and only uric acid was significantly abnormal in the blood test results (blood uric acid, 577 μmol/L). On the day of treatment, the patient's uric acid level remained high (blood uric acid, 662 μmol/L). Subsequently, we performed a musculoskeletal ultrasound. Given the age of the patient, it was not possible to determine whether this echo signal was the sodium urate crystallization or costal cartilage calcification. Therefore, we performed the dual-energy CT scan of the patient's chest. we found many sodium urate crystals in the patient's costal cartilage according to dual-energy CT scanning. To reduce the impact of false positives ^[1]^, the screening parameters were Rho > 120, 7 < Z_eff_ < 8, and there were still flares near the sternum. The preliminary diagnosis was costochondral gout.

The patient complained of no pain other than in the chest, which is a rare site of gout. In previous case reports, this symptom usually occurred in conjunction with other typical gout sites and few isolated cases were reported ^[2]^. Therefore, we performed further tests on the patient's hands and feet. To our surprise, the dual-energy CT scans showed no sodium urate crystals on either his hands or feet.

The patient reported that he had taken Aceclofenac (200 mg/day) for two days before the visit, but the symptoms of chest pain did not relieve. Then the Etoricoxib (120 mg/day) was advised for pain relief, but the pain did not relieve after one day in drug taking. Benzbromarone (100 mg/day) benzbromarone was added to prevent future flares and to shrink the tophaceous deposit in the costochondral region. After two days of combined therapy, the pain quickly subsided. the patient was treated with Febuxostat (40 mg/day) for a long period of time after the pain relief, treated with the Febuxostat for 2 weeks, the patient's blood uric acid level decreased to 317 μmol/L. No further pain was reported by the patient up to now.

Since the patient was diagnosed with hyperuricemia, the fasting blood uric acid level has remained high. The patient reported to us his daily diet, which followed the China multi-disciplinary expert consensus on diagnosis and treatment of hyperuricemia and related diseases (2023 edition). To rule out the familial inheritance of hyperuricemia, fasting uric acid records of the patient's parents and grandparents were collected in Table 1. According to the result, the possibility of familial inheritance of hyperuricemia in the patient was initially ruled out. The fasting blood uric acid test records traceable to the patient since the diagnosis of hyperuricemia are shown in Table 2.

Table 1 fasting uric acid of the patient's family members

| Character | Blood uric acid (μmol/L) |
| --- | --- |
| Father | 374 |
| Mother | 270.6 |
| Grandfather | 299 |
| Grandmother | 257 |

Table 2 Fasting blood uric acid test record of the patient

| Date (Y/M/D) | Blood uric acid (μmol/L) | Whether have gout symptoms |
| --- | --- | --- |
| 2020/5/27 | 518 | YES |
| 2022/6/17 | 506 | NO |
| 2022/6/24 | 614 | NO |
| 2022/7/1 | 626 | NO |
| 2022/7/8 | 619 | NO |
| 2022/7/15 | 612 | NO |
| 2023/9/18 | 577 | NO |
| 2023/11/16 | 662 | YES |
| 2023/12/5* | 317 | NO |

* After Febuxostat treatment.

# Discussion

In recent years, the incidence of gout is increasing gradually ^[3]^,.The onset of gout has become younger and more confined to the foot (especially the first metatarsophalangeal joint), ankle, hand and wrist joints ^[4]^. In the diagnosis of chest pain in patients with hyperuricemia, costochondral gout should also be considered as a cause. This poses a challenge for the clinical diagnosis of pain-related diseases in patients with hyperuricemia.

Analgesia should be given priority, the use of urate-lowering drugs should be avoided during the period to prevent secondary pain caused by melting crystals in the acute phase of gout, according to the guidelines^[5]^. However, combined use of urate-lowering drugs may be a better choice in the treatment of costal cartilage gout. However, it should be noted that before such treatment, the patient should be confirmed that there is no urate crystallization in the common site.

The patient was asked about a history of trauma to the anterior chest or a history of cardiopulmonary resuscitation, research given the fact that gout is more likely to develop at sites of joint injury ^[6]^. However, the patient denied any such history.

For patients with hyperuricemia who are young and have a normal BMI and no other metabolic diseases (e.g., hyperglycemia and hyperlipidemia), they are more inclined to use low-purine dietary interventions. However, the benefit of dietary interventions for hyperuricemia is limited ^[7]^.

Although synovial fluid aspiration and monosodium urate crystal analysis is the gold standard of diagnosing early-onset gout, there has been a risk of postoperative infection associated with fluid aspiration, and few adverse events have been reported in gout diagnosis with dual-energy CT and musculoskeletal ultrasonography ^[8]^. However, the problems of the two non-invasive detection methods are also obvious, there is the possibility of artifacts in the images of dual-energy CT, and the chemical composition of the lesion cannot be accurately judged by musculoskeletal ultrasound, so the two tests should be combined in the diagnosis of gout in unconventional parts.

The early onset of costochondral gout is not necessarily accompanied by redness, swelling, fever, and elevated C-reactive protein levels, which poses a challenge to the diagnosis of costochondral gout and other costochondritis.

# References

1. Yokose C, Eide SE, Huber FA, Simeone FJ, Ghoshhajra BB, Shojania K*, et al.* Frequently Encountered Artifacts in the Application of Dual-Energy Computed Tomography to Cardiovascular Imaging for Urate Crystals in Gout: A Matched-Control Study. Arthritis Care Res (Hoboken) 2024. doi: 10.1002/acr.25312.

2. Yoshida Y, Harada Y, Shimizu T. Costochondral gout. Canadian Medical Association Journal 2022;194:E1338-E1338. doi: 10.1503/cmaj.220216-f.

3. Safiri S, Kolahi AA, Cross M, Carson-Chahhoud K, Hoy D, Almasi-Hashiani A*, et al.* Prevalence, Incidence, and Years Lived With Disability Due to Gout and Its Attributable Risk Factors for 195 Countries and Territories 1990-2017: A Systematic Analysis of the Global Burden of Disease Study 2017. Arthritis & Rheumatology 2020;72:1916-1927. doi: 10.1002/art.41404.

4. Khanna P, Johnson RJ, Marder B, LaMoreaux B, Kumar A. Systemic Urate Deposition: An Unrecognized Complication of Gout? Journal of Clinical Medicine 2020;9. doi: ARTN 3204

10.3390/jcm9103204.

5. Tai V, Gow P, Stewart S, Satpanich P, Li C, Abhishek A*, et al.* An updated systematic review and meta-analysis of randomised controlled trials on the effects of urate-lowering therapy initiation during a gout flare. Semin Arthritis Rheum 2024;65:152367. doi: 10.1016/j.semarthrit.2024.152367.

6. Xu H, Qin H, Hua Y, Dalbeth N. Contributions of joint damage-related events to gout pathogenesis: new insights from laboratory research. Ann Rheum Dis 2023;82:1511-1515. doi: 10.1136/ard-2023-224679.

7. Mikuls TR. Gout. N Engl J Med 2022;387:1877-1887. doi: 10.1056/NEJMcp2203385.

8. Newberry SJ, FitzGerald JD, Motala A, Booth M, Maglione MA, Han D*, et al.* Diagnosis of Gout: A Systematic Review in Support of an American College of Physicians Clinical Practice Guideline. Annals of Internal Medicine 2017;166:27-+. doi: 10.7326/M16-0462.

# Funding

Funding provided by The Chinese National Natural Sciences Foundation (82370816)

# Competing Interests

We declare that there were no competing interests.

# Author Contributions

YZ, YH and XC contributed to the acquisition, analysis and interpretation of data for the article; YZ, DH and HZ to the interpretation of data; ZH, ⁠YZ and PL drafted and critically revised the article; TZ and YJ revised the article critically for important intellectual content;⁠ HZ, ZH and DH provided funding support. ⁠All authors approved the version to be published;⁠ ZH is the grantor of the study.

# Ethics approval

This study does not involve any research intervention on the patient and this study was approved by the Xinxiang Medical University.

# Consent to participate

Written informed consent was obtained from the patient.

# Consent to publish

Written informed consent was obtained from the patient.

# Data availability

We declare the availability of data when asked.
